# Exploiting Molecular Ions for Screening Hydrophobic Contaminants in Sediments Using Gas Chromatography-Atmospheric Pressure Chemical Ionization-Ion Mobility-Mass Spectrometry

**DOI:** 10.1021/acs.est.4c13059

**Published:** 2025-02-25

**Authors:** Xiaodi Shi, Håkon A. Langberg, Anna Sobek, Jonathan P. Benskin

**Affiliations:** † Department of Environmental Science, Stockholm University, Stockholm 10691, Sweden; ‡ Geotechnics and Environment, Norwegian Geotechnical Institute, Oslo 0484, Norway

**Keywords:** atmospheric pressure chemical ionization, collision cross section, hydrophobic contaminants, sediment, neutral per- and polyfluoroalkyl substances

## Abstract

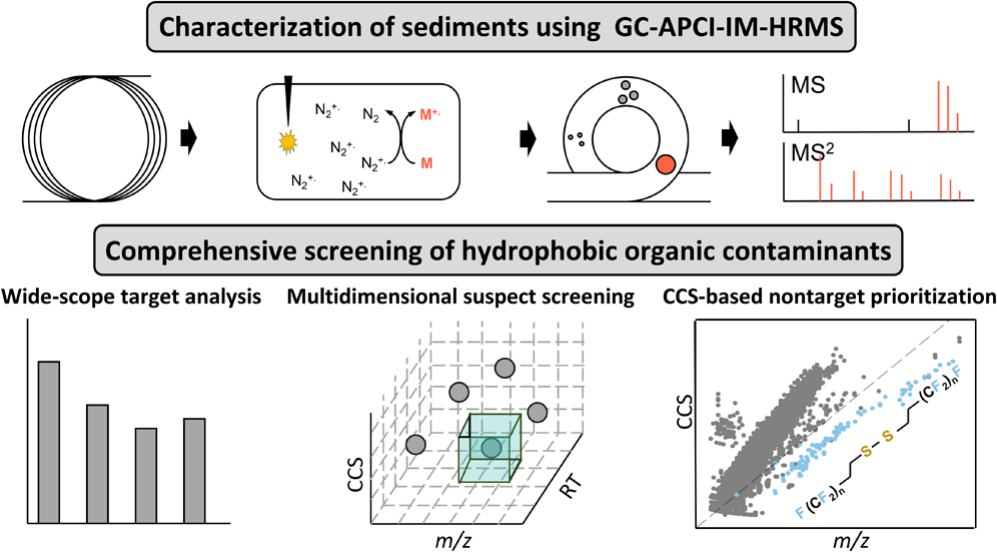

Hydrophobic organic contaminants (HOCs) are conventionally screened by matching electron ionization (EI) mass spectra acquired using gas chromatography–mass spectrometry (GC–MS) to reference spectra. However, extensive in-source fragmentation hampers de novo structure elucidation of novel substances that are absent from EI databases. To address this problem, a new method based on GC-atmospheric pressure chemical ionization (APCI) coupled to ion mobility-high resolution mass spectrometry (IM-HRMS) was developed for simultaneous target, suspect, and nontarget screening of HOCs. Of 102 target chemicals, 85.3% produced (quasi-)molecular ions as base peaks, while 71.6% displayed method detection limits lower than those of GC-EI-low resolution MS. The optimized method was applied to standard reference sediment and sediments from the Baltic Sea, an Arctic shelf, and a Norwegian lake. In total, we quantified 56 target chemicals with concentrations ranging from 4.86 pg g^–1^ to 124 ng g^–1^ dry weight. Further, using a combination of full scan mass spectrum, retention time, collision cross section (CCS), and fragmentation spectrum, a total of 54 suspects were identified at Confidence Level (CL) 2. Among the remaining features, 169 were prioritized using a halogen-selective CCS cutoff (100 Å^2^ + 20% mass), leading to annotation of 54 substances (CL ≤ 3). Notably, a suite of fluorotelomer thiols, disulfides, and alkyl sulfones were identified in sediment (CL 1–2) for the first time. Overall, this work demonstrates the potential of GC-APCI-IM-HRMS as a next-generation technique for resolving complex HOC mixtures in environmental samples through exploitation of molecular ions.

## Abstract 2

Hydrophobic contaminants in sediment can be comprehensively screened using a combination of soft ionization to preserve molecular ions and ion mobility to derive collision cross sections.

## Introduction

Chemical pollution is recognized as one of nine planetary boundaries which threaten the stability of Earth systems.^1^ Between 700 and 1700 new chemicals are registered in the United States and European Union every year,^2^ and many more remain undocumented until they are discovered in the environment. This situation challenges current monitoring frameworks to move beyond characterization and risk assessment of single chemicals, toward quantitative screening of as many known substances as possible, while simultaneously collecting nontarget data suitable for identifying unknown compounds.^3^

Prior screening work with liquid chromatography-electrospray ionization-high resolution mass spectrometry (LC-ESI-HRMS) has achieved considerable success in detecting and identifying emerging chemicals.^3^ However, LC-based methods tend to be limited to substances with polar functional groups that are ionizable by ESI. Outside of this chemical space fall hydrophobic organic contaminants (HOCs), which are problematic due to their tendency to accumulate in organic carbon (OC) and lipids.^3,4^ Typically, HOCs are analyzed using gas chromatography–electron ionization-mass spectrometry (GC-EI-MS). The reproducible fragmentation obtained from EI at 70 eV enables annotation of known HOCs through matching of experimental to reference mass spectra.^5^ However, the frequent absence of molecular ions in EI impedes de novo structural elucidation of unknown compounds.^6^ While electron capture negative ionization (ECNI) offers a partial solution to this problem, this technique tends to be too selective for broad screening, since only a small group of compounds with a resonance structure and high electron affinity can generate molecular ions.^7,8^ Therefore, an ionization technique for GC that is both universal and “soft” is critical for comprehensive screening of HOCs.

Atmospheric pressure chemical ionization (APCI) is one of the few “soft”, non-selective, and commercially available ionization approaches for GC.^9−11^ APCI can efficiently preserve (quasi-)molecular ions of GC-amenable compounds through charge/proton transfer.^9^ The advantages of harnessing molecular ions have been demonstrated in studies involving wide-scope quantification of emerging contaminants and even identification of unknown compounds.^12−15^ When coupled to ion mobility (IM), instrument-independent and reproducible collision cross section (CCS) values can be acquired to provide additional structural information, improving confidence in identification.^16^ A number of pioneering studies have already used the GC-APCI-IM-HRMS to develop HOC databases and nontarget prioritization strategies based on CCS.^17,18^ However, the potential of this technique for simultaneous target, suspect, and nontarget analysis of environmental contaminants has not been fully explored.

In this study, we developed and validated a new analytical workflow for simultaneous target, suspect, and nontarget screening of HOCs in sediment samples using multidimensional information obtained from (quasi-)molecular ions. As a sink for organic carbon, sediment is a complex matrix containing a diverse range of HOCs, making it ideally suited for testing the performance of new analytical methods. Application of the new method to sediments from a diverse range of locations demonstrated its widespread applicability, and at the same time generated new quantitative and qualitative information on legacy, emerging, and even unknown substances.

## Materials and Methods

### Instrumental Analysis

A Waters quadrupole-cyclic ion mobility-time-of-flight mass spectrometer (Q-cIM-ToF; Waters Corp., Wilmslow, U.K.) coupled to an Agilent 8890 GC (Agilent Technologies, Santa Clara, CA, U.S.A) via an APCI source was employed for analysis. Sample volumes of 1 μL were injected in pulse splitless mode with a programmed inlet temperature for vaporization which was initially set at 100 °C for 0.15 min, then increased at 600 °C min^–1^ to 280 °C, and then held for 1 min. Analytes were separated using a 30 m DB-5MS Ultra Inert column (i.d., 0.25 mm; film thickness, 0.25 μm; Agilent Technologies) with helium carrier gas at a constant flow of 1.5 mL min^–1^. The GC oven temperature program started at 70 °C for 1 min, followed by an increase at 10 °C min^–1^ to 310 °C, at which point the temperature was held for 15 min.

The GC–MS interface and ion source were maintained at 290 and 150 °C, respectively. The positive ionization mode of APCI was employed with the corona discharge and cone voltage at 2 μA and 30 V, respectively. N_2_ was used as the makeup gas, auxiliary gas, and cone gas at flow rates of 200 mL min^–1^, 350 L h^–1^, and 250 L h^–1^, respectively, in the dry condition. Potential moisture in the makeup gas was removed by a gas purifier (VICI Mat/Sen Purifier Module, Nitrogen, RESTEK). An uncapped bottle of water was placed in the ionization enclosure to achieve wet conditions. Auxiliary and cone gas flow rates were reduced to 150 and 200 L h^–1^, respectively, under wet conditions. The ion source was conditioned overnight before use. The negative ionization mode of APCI is similar to ECNI and only selective to electrophilic compounds.^11^ Therefore, the negative ionization mode was not used.

The MS was operated in high-definition MS^E^ mode with a mass range of 100–1200 Da. Collision energy was fixed at 6 eV in low energy mode and ramped between 15 and 50 eV in high energy mode, with a scan time of 0.3 s for each mode. The cIM cell was operated in one pass mode with 3 pushes per bin at a traveling wave height of 22 V. N_2_ was used for both drift and collision gases. Column bleeding (C_9_H_27_O_5_Si_5_^+^: mass-to-charge ratio [*m*/*z*] 355.0705) was measured every 2 min for internal mass correction. CCS was calibrated using a mixture of 22 compounds supplied by Waters Corp., following their standard procedure. Tuning parameters are listed in the Table S1 in the Supporting Information Excel file.

### Optimization of Instrumental Parameters

For the above method, APCI source parameters were optimized to minimize in-source fragmentation and enhance sensitivity. This included auxiliary gas, cone gas, makeup gas, corona current, and cone voltage under both wet and dry conditions. The inter- and intraday variability in ionization pathways (i.e., charge- and proton transfer) under dry and wet conditions were also evaluated. Given that HOCs often contain halogen atoms which increase their molecular weight, tuning parameters were optimized based on ions generated from column bleeding at 70 °C to increase intensities of ions with higher *m*/*z*. Optimization results are shown in the Section A and Figures S1–S5 in the Supporting Information.

### Sample Collection and Preparation

To demonstrate the widespread applicability and robustness of our method, a diverse set of sediments were analyzed, each influenced by different sources of contamination and containing different concentrations of OC. Surface marine sediments from the Baltic Sea (9 sites; 0–2 cm; 1.6–7.7% OC) were impacted by both local sources and long-range transport. Surface lake sediment from Lake Tyrifjorden, Norway (1 site; 0–2 cm; 4.5% OC) received emerging contaminants (e.g., per- and polyfluoroalkyl substances [PFAS]) primarily from a local paper production plant.^19^ For marine sediments from the remote East Siberian Sea (2 sites; 0–1 cm; 0.94–1.4% OC), we expected low concentrations of pollutants, primarily associated with long-range transport and deposition.^20^ NIST standard reference material (SRM; 1941b-Organics in Marine Sediment; 2.99% OC) was also included for method validation. Detailed sampling information and total organic carbon content (TOC) for all sediment samples are provided in Section B and Table S2 of the Supporting Information.

The extraction method was modified from U.S. Environmental Protection Agency Method 3545A.^21^ Approximately 4 g of each freeze-dried sediment was fortified with 28 isotope-labeled internal standards (IS; listed in Table S3 in the Supporting Information Excel file) and extracted using an accelerated solvent extraction system (ASE 350; Dionex, U.S.A) three times with 20 mL of acetone/*n*-hexane (1:1 v/v) per cycle. The static state was held at 100 °C for 10 min. After rotary evaporation, sulfur was removed using activated copper. Because of reduced fragment ions and lowered baseline using APCI and HRMS, no further cleanup was conducted in order to enhance throughput and avoid inadvertent removal of nontarget substances. Finally, extracts were concentrated to approximately 200 μL under a nitrogen stream. Further details on the sample preparation method are provided in Section C of the Supporting Information. All glassware and quartz filters were burned at 450 °C for 4 h, and metal parts were ultrasonicated for 20 min using acetone. Except for PEEK seals for ASE and caps for sample vials, no other plastic items were used for sample preparation. To prevent potential photolysis, containers were either wrapped in aluminum foil or amber glassware was used.

### Method Validation and Quality Control

Analytical performance of the method was evaluated using 102 environmental contaminant standards (1.44< log *K*_ow_ < 16.8; −9.31< log *K*_aw_ < 11.3; Table S3 in the Supporting Information Excel file), including polycyclic aromatic hydrocarbons (PAHs), organophosphate esters (OPEs), polychlorinated biphenyls (PCBs), polybrominated diphenyl ethers (PBDEs), PFAS, and other halogenated compounds.

Accuracy and precision of target HOC analysis was evaluated in two ways: First, by comparing triplicate measurements of SRM 1941b to certified concentrations; and second, via spike/recovery experiments with Baltic Sea sediment (58.26° N, 16.91° E), which was fortified in triplicate to ∼1 ng g^–1^ dry weight (dwt) with a standard of each of the 102 targets. Percent recoveries were calculated after subtracting background intensities measured in unfortified sediment. In addition, variability in relative ionization efficiencies were calculated based on measurements within 1 day and across different days. Lab contamination was monitored via procedural blanks (one per batch) using diatomaceous earth (DE; Dionex, Thermo Scientific) as received, which underwent the same extraction process as sediments. Since intensities in blanks were negligible compared to those in real samples, blank subtraction was not performed. Instrumental detection limits (IDLs) were estimated based on standard deviations (SD) of peak area from five replicate measurements of contaminant mixtures at the lowest detectable concentrations, while method detection limits (MDLs) were the sum of concentrations in procedural blanks and SD of peak area using triplicates of fortified sediment. Concentrations lower than MDLs were flagged in the target analysis. For suspect and unknown compounds, relative intensities in samples lower than the average plus three times the SD of those in procedural blanks were considered not detected.

For comparison of method sensitivity, IDLs and MDLs were also determined using a low-resolution ISQ GC-EI-MS (Thermo Scientific, U.S.A) and an 8890 GC-ECNI-5977B MS (Agilent Technologies), both in selected ion monitoring (SIM) mode. Details of these methods are provided in Section D in the Supporting Information and Table S3 in the Supporting Information Excel file.

### Target Analysis

We quantified 102 HOCs using reference standards. The most intense isotopic ion under either dry (typically M^+•^) or wet (typically [M + H]^+^) conditions was selected as the quantitative ion. All selected ions are listed in Table S3 in the Supporting Information Excel file, along with their preferred source conditions. After integration using Masslynx (version 4.2, Waters Corp.), targets were quantified using a linear relative response-based calibration curve (i.e., native/IS) with 1/*x* weighting.

### Suspect Analysis

Full scan (i.e., MS^1^) and fragmentation (i.e., MS^2^) spectra were exported separately from Progenesis QI (version 3.0, Waters Corp.), following lockmass correction, pairing of precursor and product ions, peak picking, and alignment. We retained peaks with areas >3 times higher than those in procedural blanks for screening. Software parameters are provided in Section E in the Supporting Information.

A multidimensional-constrained suspect screening method was employed as previously described.^22^ Briefly, two suspect lists were compiled with information on identity, exact mass, CCS, and retention time (RT). The first comprised 1060 GC-amenable compounds with experimentally derived CCS values, previously reported in the literature.^16,23^ The second included 530 chemicals derived from the Arctic Monitoring and Assessment program’s (AMAP’s) list of chemicals of emerging Arctic concern and the European Chemicals Agency’s (ECHA’s) list of substances of very high concern.^24,25^ Peaks were matched against suspect lists by *m*/*z*, RT, and CCS. MS^2^ similarity scores for candidates with matches in all three dimensions were further evaluated using SIRIUS + CSI:FingerID (version 5.8.6).^26^ Thereafter, multidimensional scores were calculated using eqs 1–4.
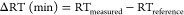
1
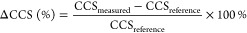
2
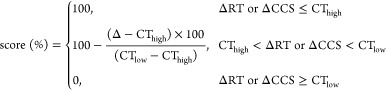
3
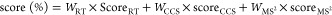
4

High- and low-confidence thresholds (CT_high_ and CT_low_) for putative compound identification were selected based on comparisons of measured and reference (literature/model-predicted) values in suspect lists. W denotes the weight for each dimension. The true positive rate was independent of the assignment of weights in this study, based on testing with real sediment samples. Considering the dependence of RT on chromatographic conditions, less weight was assigned to the RT score (*W*_RT_ = 0.2) than CCS and MS^2^ scores (0.4 for both). Peaks with a multidimensional score >60% were potential candidates, among which, the highest scoring peak was reported as putatively identified (Confidence Level [CL] 2 according to the Schymanski scale^27,28^). Details regarding the parameter assignments and model validation were described elsewhere.^22^

### Nontarget Analysis

Previous measurements and model predictions suggested that halogenated compounds tend to have smaller CCS values compared to nonhalogenated compounds with similar mass, potentially due to the greater mass density of halogen atoms.^17,29^ In this study, peaks with CCS values lower than a proposed CCS boundary (i.e., 100 Å^2^ + 20% mass) were preliminarily prioritized as halogenated compounds.^14^ Molecular compositions were determined with the number of halogen atoms constrained by isotopic patterns. Compounds at CL 1 were confirmed with a standard, while compounds classified as CL 2 were putatively identified with multidimensional and diagnostic information, according to the Schymanski scale.^27,28^ SIRIUS + CSI:FingerID was used for in silico interpretation.^26^ The highest CSI:Finger ID scoring candidate for each peak was retained and categorized as CL 3. For the remaining peaks identified at CL4, we only assigned a formula if the substance appeared to be an analogue of a substance previously identified at CL1-3. Using APCI, M^+•^, [M + H]^+^, or in-source fragment ions may occur. To determine which species were present, M^+•^ ions were identified using the nitrogen rule, while [M + H]^+^ ions were distinguished from in-source fragment ions by scrutinizing the [M + 1]^+^ to M^+•^ ratios in MS^1^ spectra acquired under both wet and dry conditions. No further efforts were made to find out which group/atom was lost for in-source fragment ions, which accounted for 52% of features at CL4.

### Semiquantification

For confirmation, 8:2/8:2 fluorotelomer disulfide was purchased from AKos GmbH (Lörrach, Germany). Its ^1^H nuclear magnetic resonance (NMR) spectrum is provided in Figure S6 in the Supporting Information. 10:2 and 12:2 fluorotelomer methyl sulfones were custom synthesized from Chiron (Trondheim, Norway).^30^ These neutral PFAS standards were used to semiquantify their homologues and analogues.^31^ Specifically, fluorotelomer thiols and disulfides were semiquantified using the mole ionization efficiency of 8:2/8:2 fluorotelomer disulfide. Average mole ionization efficiencies of 10:2 and 12:2 fluorotelomer methyl sulfones were used to semiquantify other fluorotelomer alkyl sulfones. The intrahomologue uncertainty was estimated to be 40.6%, through comparing quantified concentrations of 10:2 and 12:2 fluorotelomer methyl sulfones and semiquantified concentrations using their average mole ionization efficiency.

For suspect and unknown compounds, relative intensities (g^–1^ dwt) were calculated by normalizing the area of the compound to the dry weight of the extracted sediment and the area of 1 ng IS (i.e., ^13^C_12_-PCB47 and *d*_15_-triphenylphosphate for the dry and wet conditions, respectively). Concentrations were not calculated for these substances.

## Results and Discussion

### Method Performance

**Figure 1:**
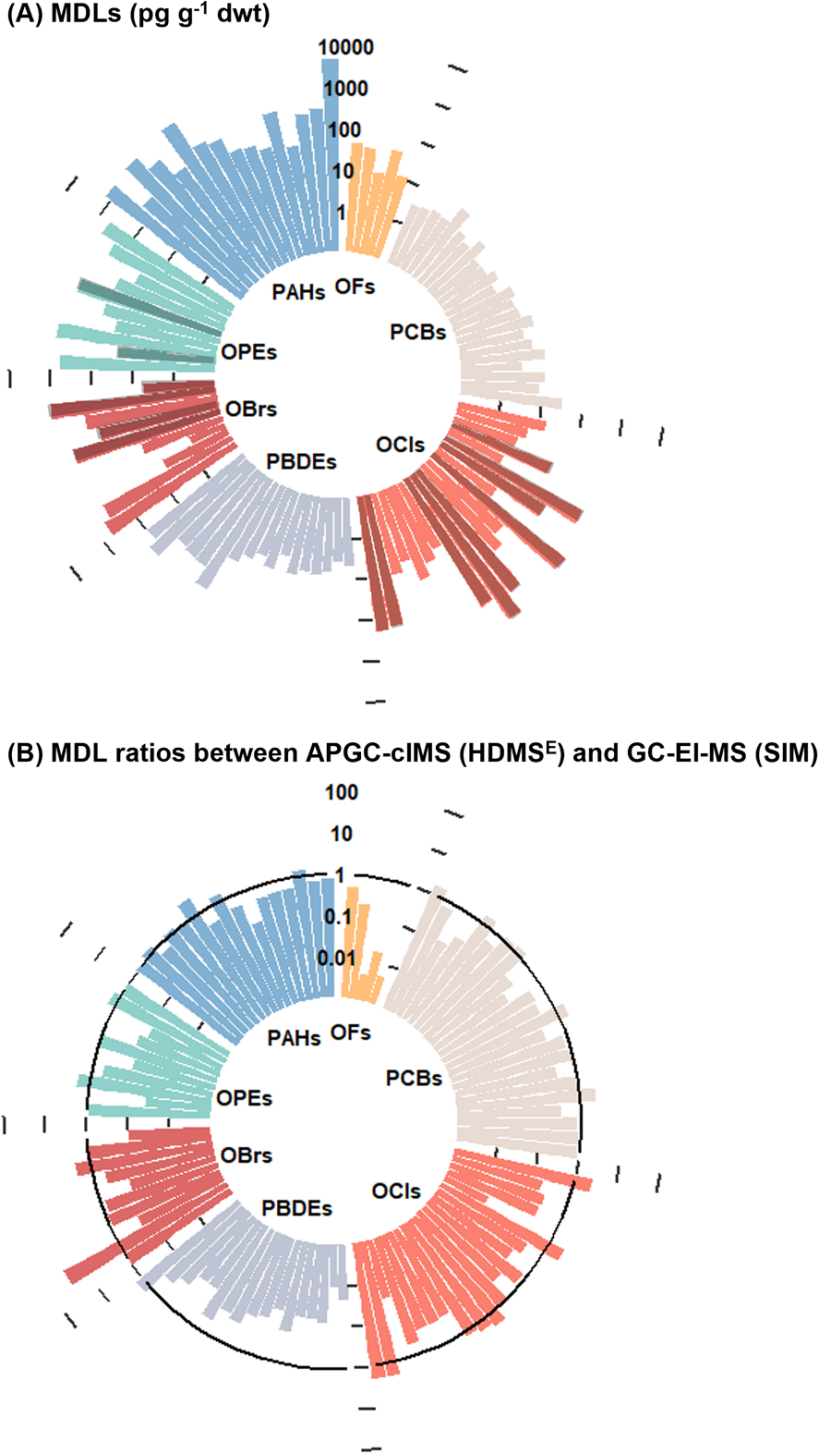
(A) Method detection limits (MDLs) using gas chromatography-atmospheric pressure chemical ionization-cyclic ion mobility-mass spectrometry (APGC-cIMS) in high definition MS^E^ mode (HDMS^E^) and (B) MDL ratios between APGC-cIMS in HDMS^E^ and gas chromatography-electron ionization-mass spectrometry (GC-EI-MS) in selective ion monitoring mode (SIM). Light bars in the panel A indicate (quasi-)molecular ions (i.e., M^+•^, [M ± H]^+^, and [M – X]^+^) as base peaks. Bars within the circle in the panel B indicate better sensitivity using the current method. OFs: organofluorines, PCBs: polychlorinated biphenyls, OCls: organochlorines, PBDEs: polybrominated diphenyl ethers, OBrs: organobromines, OPEs: organophosphate esters, and PAHs: polycyclic aromatic hydrocarbons.

Among 102 target chemicals, 85.3% produced (quasi-)molecular ions (i.e., M^+•^, [M ± H]^+^, and [M – X]^+^) as base peaks (Figure 1A), indicating the softness of this technique for HOCs. Specifically, aromatic compounds, such as PAHs, PCBs, PBDEs, and halogenated benzenes, were ionized through charge transfer without fragmentation (i.e., M^+•^). Quasimolecular ions (i.e., [M – H]^+^ or [M – Br]^+^) were base peaks for semifluorinated alkanes, two organic chlorinated pesticides, and two brominated flame retardants. Although some compounds fragmented in the source, the number of fragments was limited, particularly compared to using EI (see Figure S1 in the Supporting Information for a representative MS^1^ and Table S3 in the Supporting Information Excel file for details of in-source fragmentation). Under wet conditions, [M + H]^+^ was predominantly the base peak for oxidized compounds and OPEs. In comparison, only molecular ions of aromatic compounds were quantitative ions using EI, while only 68 halogenated compounds were ionized using ECNI, primarily with halogen ions as base peaks (Table S3 in the Supporting Information Excel file). These results suggest that APCI is a universal ionization technique for a wide range of known HOCs and that it also preserves molecular ions necessary for unknown identification.

Owing to reduced fragmentation and lower baselines using APCI and HRMS, MDLs for 71.6% of target compounds using the current technique were better than those achieved with GC-EI-MS in SIM mode (Figure 1B). Notably, semifluorinated alkanes and OPEs displayed orders of magnitude lower MDLs using GC-APCI compared to GC-EI, likely due to reduced fragmentation using the former approach. IDLs and MDLs using different methods for each compound are listed in Table S3 in the Supporting Information Excel file.

IS-corrected recoveries for 67.6% of the target chemicals fell within the range of 70–130% with a SD < 30% (for compound-specific recoveries, see Table S3 in the Supporting Information Excel file). The remaining compounds were mostly comprised of semifluorinated alkanes and PBDEs. Low recoveries for these substances are likely associated with the high volatility of semifluorinated alkanes and the susceptibility of PBDEs to thermal degradation.

Among our target compounds, concentrations of 15 PAHs, 14 PCBs, and 3 organochlorine pesticides were consistent with certified concentrations in SRM 1941b, with an average accuracy of 103% ± 31.6% (Figure S7 in the Supporting Information). Inter- and intraday variability in relative ionization efficiencies were 12.5% and 11.0%, respectively (Table S3 in the Supporting Information Excel file for compound-specific values). Overall, the high sensitivity, recovery, and accuracy of the method for a diverse range of HOCs demonstrate its potential as a reliable technique for comprehensive contamination assessment in sediments.

### Wide-Scope Quantification of Chemicals in Sediment

**Figure 2:**
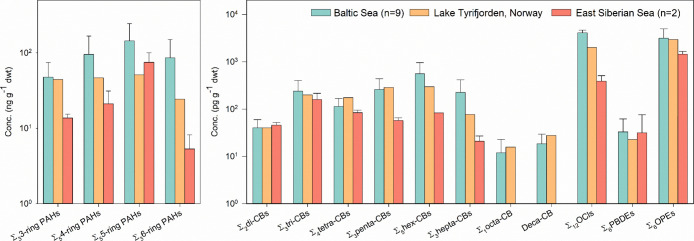
Average concentrations and standard deviations of contaminants in sediments from different locations. PAHs: polycyclic aromatic hydrocarbons, CB: chlorinated biphenyls, OCls: organochlorines, PBDEs: polybrominated diphenyl ethers, and OPEs: organophosphate esters.

Application of the optimized method to unfortified marine and lake sediments revealed 56 quantifiable substances observed in at least 1 sample, including PAHs, PCBs, PBDEs, organochlorines, and OPEs (Figure 2). Concentrations for individual compounds cover 5 orders of magnitude, ranging from 4.86 pg g^–1^ dwt to 124 ng g^–1^ dwt (concentrations are provided in Table S4 of the Supporting Information Excel file). Sediment from the Baltic Sea contained the highest level of contamination (Figure 2). In contrast, contamination in Arctic sediment, representing a remote area with little human activity, was about six and three times lower than that of Baltic Sea and Lake Tyrifjorden sediments, respectively.

HOC concentrations observed in sediments from the present work are comparable to previously reported concentrations. For example, average concentrations of Σ_16_PAHs and Σ_7_PCBs in sediment from the Swedish Baltic Sea coast (336 and 0.95 ng g^–1^ dwt, respectively) are consistent with prior measurements from the same region (i.e., Σ_16_PAHs: 157–502 ng g^–1^ dwt and Σ_7_PCBs: 0.73–2.48 ng g^–1^ dwt).^32^ Similarly, for Arctic samples, average concentrations of Σ_16_PAHs (45.0 ng g^–1^ dwt) and Σ_7_PCBs (209 pg g^–1^ dwt) in the present work align with prior reports from the Chukchi Sea (14.4–218 ng g^–1^ dwt for Σ_16_PAHs; 40.2 pg g^–1^ dwt Σ_7_PCBs)^33^ and East Siberian rivers (Σ_7_PCBs: 214–328 pg g^–1^ dwt).^34,35^

OPEs, used as flame retardants, plasticizers, and additives, have become an emerging concern due to their increasing production after the effective regulation of PBDEs.^36^ Their concentrations and relative contributions tend to vary with usage and proximity to source. In this study, average concentrations of Σ_5_OPEs were 3.17 and 2.95 ng g^–1^ dwt in Baltic Sea and Lake Tyrifjorden sediment, respectively, with tris(2-ethylhexyl) phosphate as the predominant compound. Triphenylphosphate, tris(2-butoxyethyl) phosphate, and 2-ethylhyexyl diphenyl phosphate were previously quantified in sediment and other matrices from the Arctic.^37^ Particularly, the concentration of triphenylphosphate (79.7 pg g^–1^ dwt) is close to that reported in a previous study in the Chukchi Sea (not detected-102 pg g^–1^ dwt).^38^ Overall, these results further highlight the sensitivity and robustness of the current method for a wide range of contaminants and concentration levels thereof.

### Multidimensional-Constrained Suspect Screening

**Figure 3:**
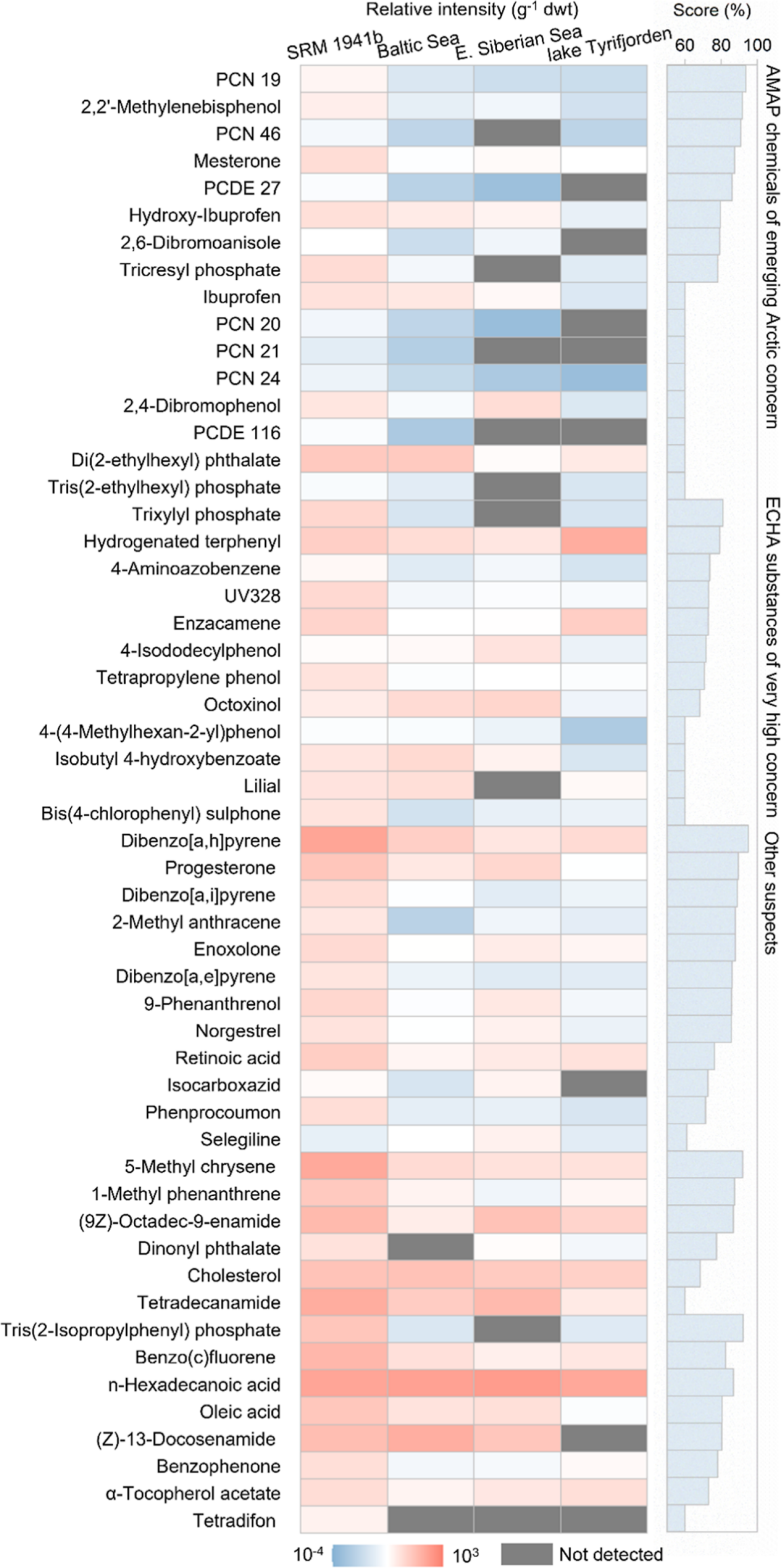
Relative intensities and multidimensional scores of suspect contaminants in sediment samples from different locations. PCN: polychlorinated naphthalene, PCDE: polychlorinated diphenyl ether, AMAP: Arctic monitoring and assessment program, and ECHA: European chemicals agency.

A total of 54 suspects were putatively identified, with multidimensional scores >60% (CL 2 according to the Schymanski scale), 28 of which are listed as chemicals of concern by AMAP or ECHA (Figure 3).^27,28^ Detailed identities, scores, and relative intensities are listed in Table S5 in the Supporting Information Excel file.

Of the 16 features tentatively matched to substances on the AMAP chemicals of emerging Arctic concern database, 11 were detected in the East Siberian Sea sediment, including trichloronaphthalenes, 2,3′,6-trichlorodiphenyl ether, di(2-ethylhexyl) phthalate, and 2,2′-methylenebisphenol (Figure 3). Given the remoteness of the Arctic from major urban settlements and chemical use, these results emphasize the persistence and long-range transport potential (LRTP) of these substances. An additional 34 compounds, found in the Arctic, were not listed in the AMAP chemicals of emerging Arctic concern database (Figure 3). Some of these compounds showed similar relative intensities across samples, indicating potential natural sources. Notably, 13 substances exhibited relative intensities in remote Arctic sediment that were orders of magnitude lower than those in samples close to human activities, suggesting long-range transport to these sites. Particularly, according to estimates using the OECD screening tool, transfer efficiencies for dibenzopyrene isomers (5.38–8.77%), bis(4-chlorophenyl) sulphone (6.42%), and UV-328 (12.4%) were above reference LRTP criteria (2.25%).^39^ Bis(4-chlorophenyl) sulphone is used in high temperature plastics, while UV-328 is a UV absorber in plastic applications. Both were identified as persistent, bioaccumulative and toxic substances by ECHA, and UV-328 is under investigation for inclusion in the United Nations Stockholm Convention on Persistent Organic Pollutants. Additionally, high relative intensities of enzacamene, octoxinol, tetrapropylene phenol, and isobutyl 4-hydroxybenzoate were observed in this study (Figure 3). All of these compounds are listed as ECHA substances of very high concern, due to their potential endocrine disrupting properties.

### CCS and *m/z-*Based Prioritization of Halogenated Compounds

**Figure 4:**
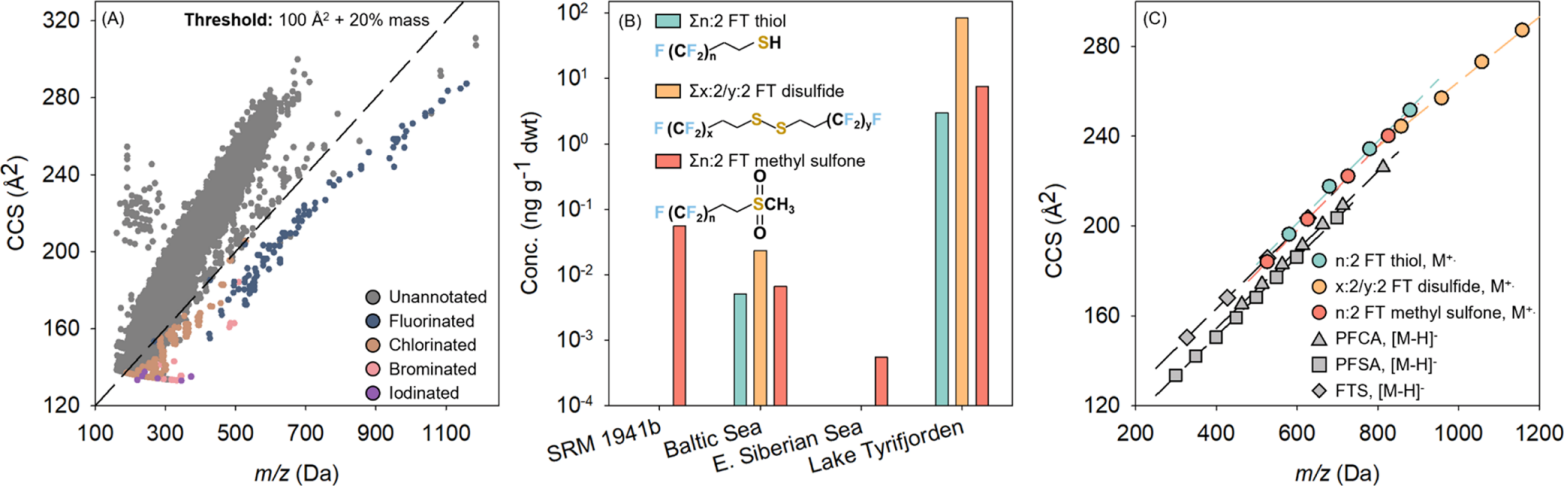
(A) Mass-to-charge ratios (*m*/*z*) and collision cross section (CCS) values of peaks detected in Lake Tyrifjorden, Norway to prioritize halogenated organic compounds. (B) Semiquantified concentrations of neutral fluorotelomer (FT) substances in sediments from different locations. (C) Trends between *m*/*z* and CCS values of organofluorines. In panel A, peaks with a CCS value under the threshold (100 Å^2^ + 20% mass; dashed line) were preliminarily prioritized as potential halogenated compounds.^17^ In panel C, CCS values for perfluoroalkyl carboxylic acids (PFCA), perfluoroalkyl sulfonate acids (PFSA), and fluorotelomer sulfonates (FTS) were obtained from Dodds et al.^43^

In addition to screening of compounds with a known structure, IM-derived CCS offers a novel size dimension for prioritization of unknown compounds, especially halogenated organic compounds.^17^ The top 50 potential halogenated compounds with the highest intensity in each of four geographical regions and two source conditions were investigated after prioritization. In total, we obtained elemental compositions for 172 peaks occurring in at least one sample, detailed in Table S6 in the Supporting Information Excel file. Of these peaks, 23 were confirmed using reference chemical standards, 22 were assigned structures considering multidimensional or diagnostic information, and 32 were annotated using an in silico tool. These features included all organofluorines, -chlorines, -bromines, and -iodines (Figure 4A).

The major halogenated compounds detected by CCS and *m*/*z*-based prioritization in SRM 1941b were anthropogenic chlorinated compounds. In addition to PCB congeners and chlorinated benzenes, higher relative intensities of potential dichlorodiphenyl sulfone isomers and polychloronaphthalene congeners were also observed. In contrast, halogenated compounds in Arctic sediment were primarily naturally occuring (e.g., bromophenolic compounds and halogenated carbazoles). These substances share similar structures with anthropogenic bioaccumulative contaminants and could pose a risk to biota.^40^ The sediment from the Baltic Sea contained a mixture of anthropogenic and natural halogenated compounds.

Notably, 34 unknown fluorinated features with high relative intensities were flagged in Lake Tyrifjorden sediment using CCS and *m*/*z*-based prioritization (Figure 4A). Among them, 15 were in-source fragments, while the remainder was molecular ions, all of which contained homologue series. The elemental composition of potential neutral PFAS included F(CF_2_)_*n*_(CH_2_)_*x*_SH (*x* = 2, *n* = 10, 12, 14, and 16; *x* = 3, *n* = 8), F_2_(CF_2_)_*n*_(CH_2_)_*x*_S_2_ (*x* = 4, *n* = 14, 16, 18, and 20; *x* = 6, *n* = 16 and 18), F(CF_2_)_*n*_C_5_H_9_S_2_O (*n* = 8, 10, and 12), F(CF_2_)_*n*_C_3_H_7_SO_2_ (*n* = 8, 10, 12, and 14) and F(CF_2_)_8_C_4_H_7_SO_2_ (Table S6 in the Supporting Information Excel file). Among these substances, F(CF_2_)_*n*_(CH_2_)_*x*_SH homologues were putatively identified as fluorotelomer thiols due to the presence of a [M–F–SH_2_]^+^ in-source fragment (Figure S8A in the Supporting Information). Unfortunately, this assignment remains tentative (CL 2) due to the unavailability of chemical standards. F_2_(CF_2_)_*n*_(CH_2_)_*x*_S_2_ was tentatively identified as a fluorotelomer disulfide based on cleavage of the S–S bond (Figure S8B), and later confirmed with an authentic standard (i.e., 8:2/8:2 fluorotelomer disulfide). Finally, F(CF_2_)_*n*_C_3_H_7_SO_2_ and F(CF_2_)_8_C_4_H_7_SO_2_ were tentatively identified as fluorotelomer alkyl sulfones, based on loss of an alkyl group (Figure S8C,D), and were subsequently confirmed using reference standards of 10:2 and 12:2 fluorotelomer methyl sulfones.

Fluorotelomer thiols are a precursor in manufacturing fluorotelomer mercaptoalkyl phosphate esters (FTMAP), also known as S-diPAPs (trade name Lodyne P208E).^41^ These neutral PFAS were used in the food packaging industry since 1995 and their detection aligns with the known use of fluorotelomer sulfonate (FTS) precursors such as FTMAP and 3-[2-(perfluoroalkyl)ethylthio] propionate (trade name Zonyl FSA) in the paper manufacturing facility upstream of Lake Tyrifjorden.^19^ We hypothesize that the disulfide could be an impurity occurring in formulations used at the site. While the origin of the methyl suflones is likely the same, it remains unclear if these substances are transformation products or impurities in commercial formulations. Notably, 8:2 fluorotelomer methyl sulfone was detected in one of the Arctic sediment samples (Figure 4B). Previously, its homologues were detected in the blubber of killer whales from Greenland and tentatively in influent of an Italian wastewater treatment plant.^30,42^ Overall, these results underscore the widespread environmental distribution and persistence of these substances.

The semiquantified concentrations of neutral PFAS observed in all sediment samples are provided in Table S4 in the Supporting Information Excel file. Only 12:2 fluorotelomer thiol and 8:2/8:2 fluorotelomer disulfide were detected in the Baltic Sea sediment, while fluorotelomer methyl sulfones appeared in almost all samples. The sum concentration of neutral PFAS in the Norwegian lake sediment was 96.1 ng g^–1^ dwt with 88.3% attributed to fluorotelomer disulfides (Figure 4B and Table S4). This value is three times higher than the sum concentration of FTSs reported previously (30.9 ng g^–1^ dwt).^19^

CCS values of neutral PFAS observed here are linearly associated with their *m*/*z*, which has also been observed for polar PFAS measured with LC-ESI systems (Figure 4C).^43^ Due to the instrument-independence of both CCS values and *m*/*z*, these class-specific trends can be leveraged to comprehensively prioritize and identify these groups of compounds across different sample types, facilitating further investigation of their sources and health effects.

### Implications

Using GC-APCI-IM-HRMS, a powerful workflow for screening of HOCs in environmental samples was established, which enabled us to detect and/or quantify 56 target, 54 suspect, and 56 unknown compounds with CL ≤ 3 in sediment samples. A series of novel neutral fluorotelomer thiols, disulfides, and alkyl sulfones were identified (CL 1–2) in sediment for the first time along with their *m*/*z* and CCS values. This work demonstrated that GC-APCI can effectively preserve (quasi-)molecular ions for HOCs, and that IM-HRMS can acquire confirmatory CCS as a semiorthogonal dimension. In addition to wide-scope quantification and highly specific suspect screening, their combination has great potential for de novo elucidation of unknown halogenated compounds, while demonstrating the potential to repurpose in silico techniques originally intended for HPLC-based analysis for use with GC-based measurements. Therefore, GC-APCI-IM-HRMS could be a next-generation technique for analyzing HOCs in complex matrices through a thorough exploitation of molecular ions.

## Supporting Information Available

Tuning parameters (Table S1); qualitative and quantitative information on target compounds (Table S3); (semi)quantified concentrations of contaminants (Table S4); scores and relative intensities of suspected compounds (Table S5); qualitative information and relative intensities of nontarget compounds (Table S6) (XLSX)
Optimization of source and tuning parameters (Section A and Figures S1–S5); sampling and TOC information (Section B and Table S2); sample preparation (Section C); instrumental methods of GC-low resolution MS (Section D); software parameters (Section E); ^1^H NMR spectrum of 8:2/8:2 fluorotelomer disulfide (Figure S6); comparison of measured and certified concentrations of contaminants in SRM 1941b (Figure S7); representative in-source fragments of fluorotelomer thiols, disulfides, and alkyl sulfones (Figure S8) (PDF)
