# Supplementary material for: Exploiting Molecular Ions for Screening Hydrophobic Contaminants in Sediments Using Gas Chromatography-Atmospheric Pressure Chemical Ionization-Ion Mobility-Mass Spectrometry
Source: Environ Sci Technol. 2025 Feb 25;59(9):4699–708. doi: 10.1021/acs.est.4c13059 (PMC11912331; doi:10.1021/acs.est.4c13059)
Supplement: Supplementary file 2 — es4c13059_si_002.pdf [file es4c13059_si_002.pdf]

# Supporting Information

## Exploiting Molecular Ions for Screening Hydrophobic Contaminants in Sediments using Gas Chromatography-Atmospheric Pressure Chemical Ionization-Ion Mobility-Mass Spectrometry

Xiaodi Shi[1]*, Håkon A. Langberg[2], Anna Sobek[1], Jonathan P. Benskin[1]

1. Department of Environmental Science, Stockholm University, Stockholm 10691, Sweden
2. Geotechnics and Environment, Norwegian Geotechnical Institute, Oslo 0484, Norway

* Address correspondence to Xiaodi Shi: Department of Environmental Science, Stockholm University, Stockholm 10691, Sweden. E-mail: xiaodi.shi@aces.su.se. Telephone: 46-73-891 2864. ORCID: 0009-0008-4062-4009

Summary: 15 pages, texts, 6 tables, 8 figures, and references.

**Contents:**

**Table S1.** Tuning parameters for the Cyclic IMS. (Supporting Excel file)

**Section A.** Optimization of source and tuning parameters.

**Figure S1.** Mass spectra of dieldrin using electron ionization and atmospheric pressure chemical ionization.

**Figure S2.** Intensities of $M^{+\cdot}$ and ratios of $M^{+\cdot}/[M\text{-}Cl]^{+}$ for dieldrin using different source parameters.

**Figure S3.** Ratios of $[M+1]^{+}/M^{+\cdot}$ for phenanthrene at different flows of auxiliary gas and cone gas.

**Figure S4.** Inter- and intra-day variability in $[M+1]^{+}/M^{+\cdot}$ for phenanthrene under dry and wet conditions.

**Figure S5.** Intensity fold changes for polychlorinated biphenyls using optimized and default tuning parameters.

**Section B.** Sampling procedure and analysis of TOC.

**Table S2.** Sampling and TOC information.

**Table S3.** Qualitative and quantitative information of target compounds. (Supporting Excel file)

**Section C.** Sample preparation.

**Section D.** Instrumental methods of GC-low resolution MS.

**Section E.** Software parameters.

**Figure S6.** $^{1}$H NMR spectrum of 8:2/8:2 fluorotelomer disulfide.

**Figure S7.** Comparison of measured and certified concentrations of contaminants in standard reference sediment 1941b.

**Table S4.** (Semi-)quantified concentrations of contaminants in sediment samples. (Supporting Excel file)

**Table S5.** Scores and relative intensities of suspected compounds in sediment samples. (Supporting Excel file)

**Table S6.** Qualitative information and relative intensities of nontarget compounds in sediment samples. (Supporting Excel file)

**Figure S8.** Full scan spectra of representative neutral PFASs.

**References**

**Section A. Optimization of source and tuning parameters.**

Dieldrin was chosen as a model compound to optimize source parameters for the dry condition, due to the formation of both of $M^{+\cdot}$ and $[M-Cl]^+$ at the $MS^1$ level (Figure S1). The intensity of $M^{+\cdot}$ indicates the sensitivity, while the ratio of $M^{+\cdot}$ and $[M-Cl]^+$ indicates the extent of in-source fragmentation. Final parameters are 2 µA for the corona discharge, 30 V for cone voltage, 200 mL $min^{-1}$ for makeup gas, 350 L $h^{-1}$ for auxiliary gas, and 250 L $h^{-1}$ for the cone gas to reduce in-source fragmentation and increase sensitivity (Figure S2).

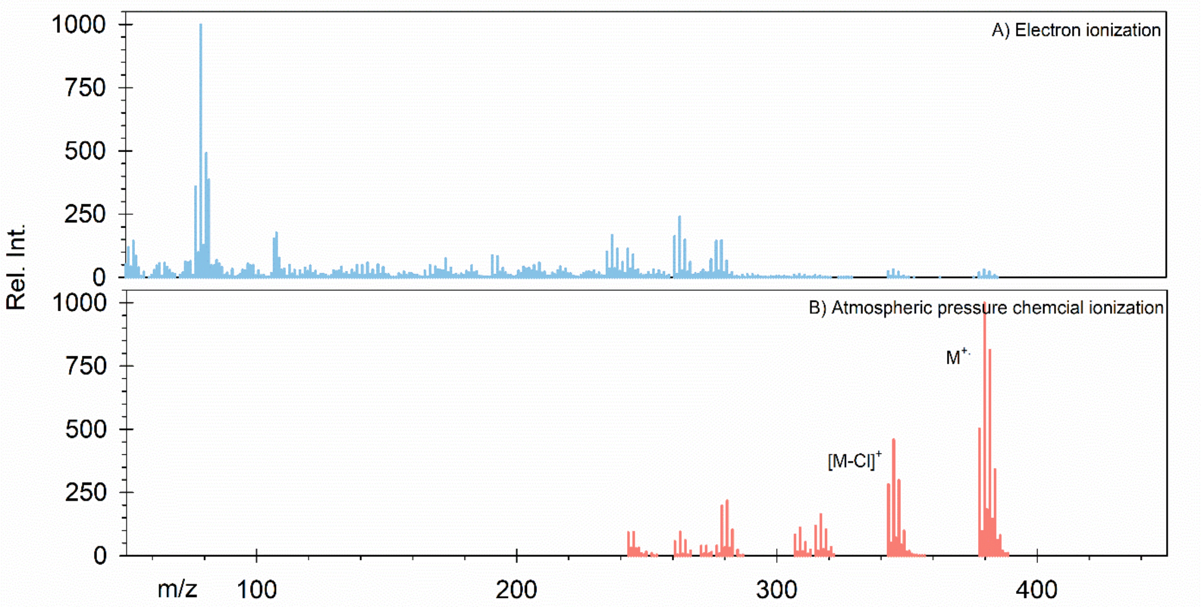


**Figure S1. Mass spectra of dieldrin using (A) electron ionization and (B) atmospheric pressure chemical ionization.**

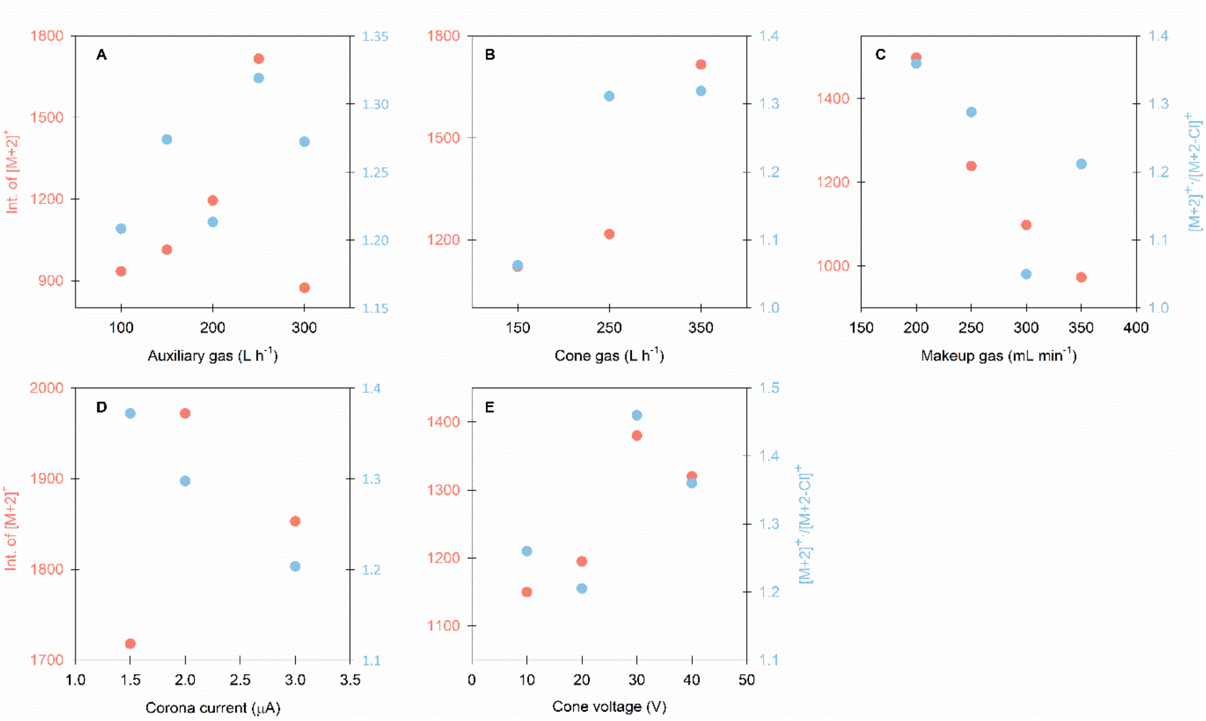


**Figure S2. Intensities of $M^{+\cdot}$ (red) and ratios of $M^{+\cdot}/[M\text{-}Cl]^{+}$ for dieldrin at different (A) auxiliary gas flow, (B) cone gas flow, (C) makeup gas flow, (D) corona current, and (E) cone voltage.**

Source parameters for the wet condition were optimized, based on those for the dry condition. Lower flows for auxiliary and cone gases are necessitated to promote protonation.[1] Since polycyclic aromatic hydrocarbons can be ionized through both charge and proton transfers, their ratios of $[M+1]^{+}$ and $M^{+\cdot}$ are typically used to indicate the extent of proton transfer.[1] This ratio for phenanthrene is shown in Figure S3. Therefore, flows for auxiliary and cone gases were reduced to 150 and 200 L $h^{-1}$, respectively. We further evaluated inter- and intra-day variability in ionization pathways (i.e., charge- and proton transfers) using the $[M+1]^{+}$ to $M^{+\cdot}$ ratio of phenanthrene (Figure S4).

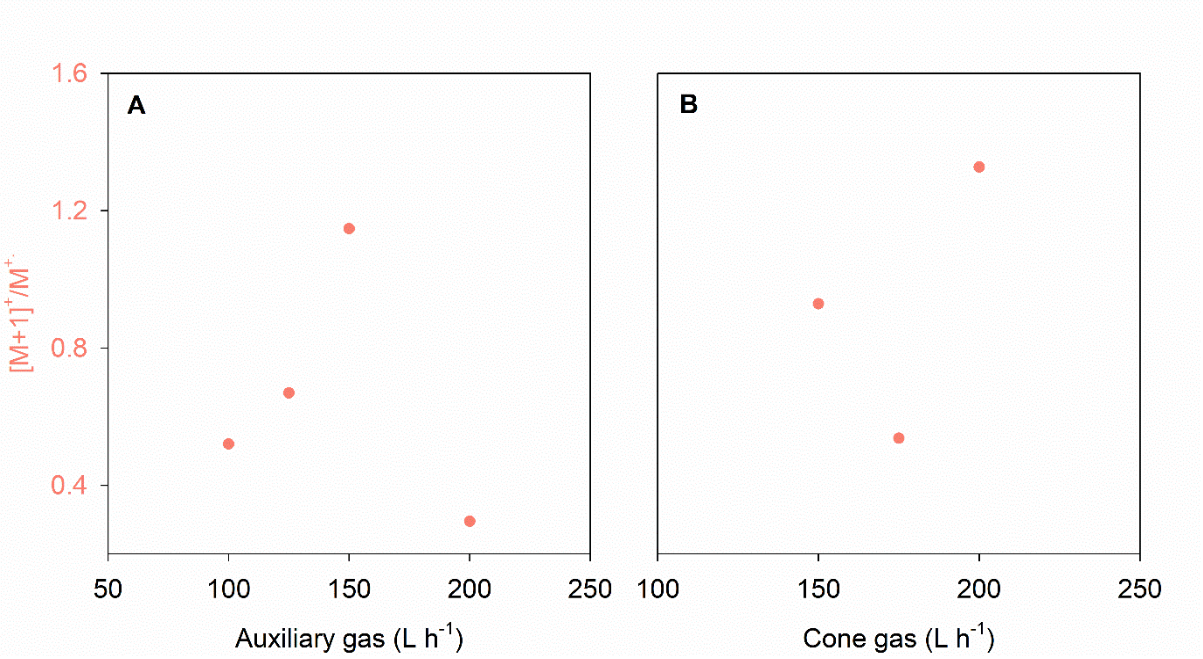


**Figure S3. Ratios of $[M+1]^+/M^{+\cdot}$ for phenanthrene at different flows of (A) auxiliary gas and (B) cone gas.**

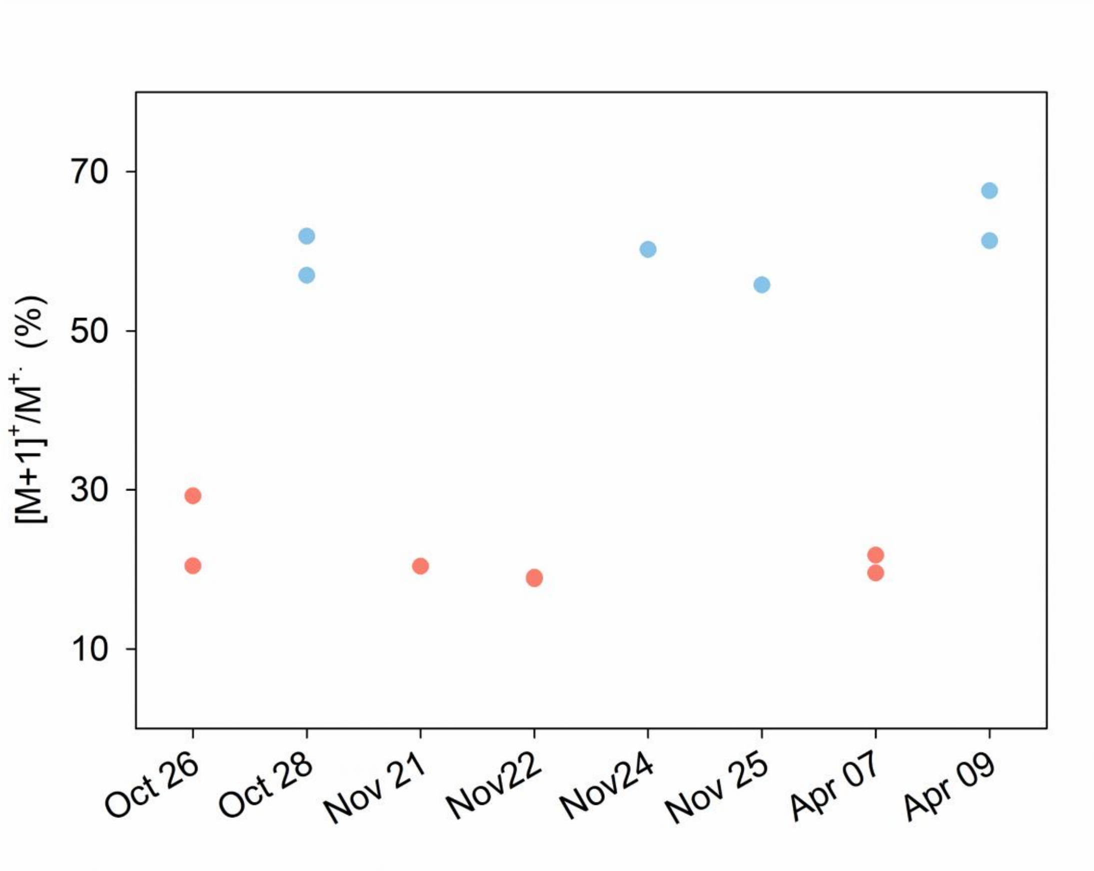


**Figure S4. Inter- and intra-day variability in $[M+1]^+/M^{+\cdot}$ (%) for phenanthrene under wet (blue dots) and dry (red dots) conditions.**

Tuning parameters for the Cyclic IMS were optimized through checking intensity variation of ions from column bleeding in tuning mode, while the GC oven maintained at 70 °C. Since HOCs are often halogenated compounds, the aim is to increase intensity of ions with higher *m/z*. The intensity fold change for polychlorinated biphenyls using optimized and default parameters is shown in Figure S5. Due to the orthogonality to source parameters, tuning parameters are identical for both dry and wet conditions. Optimized tuning parameters are listed in Table S1.

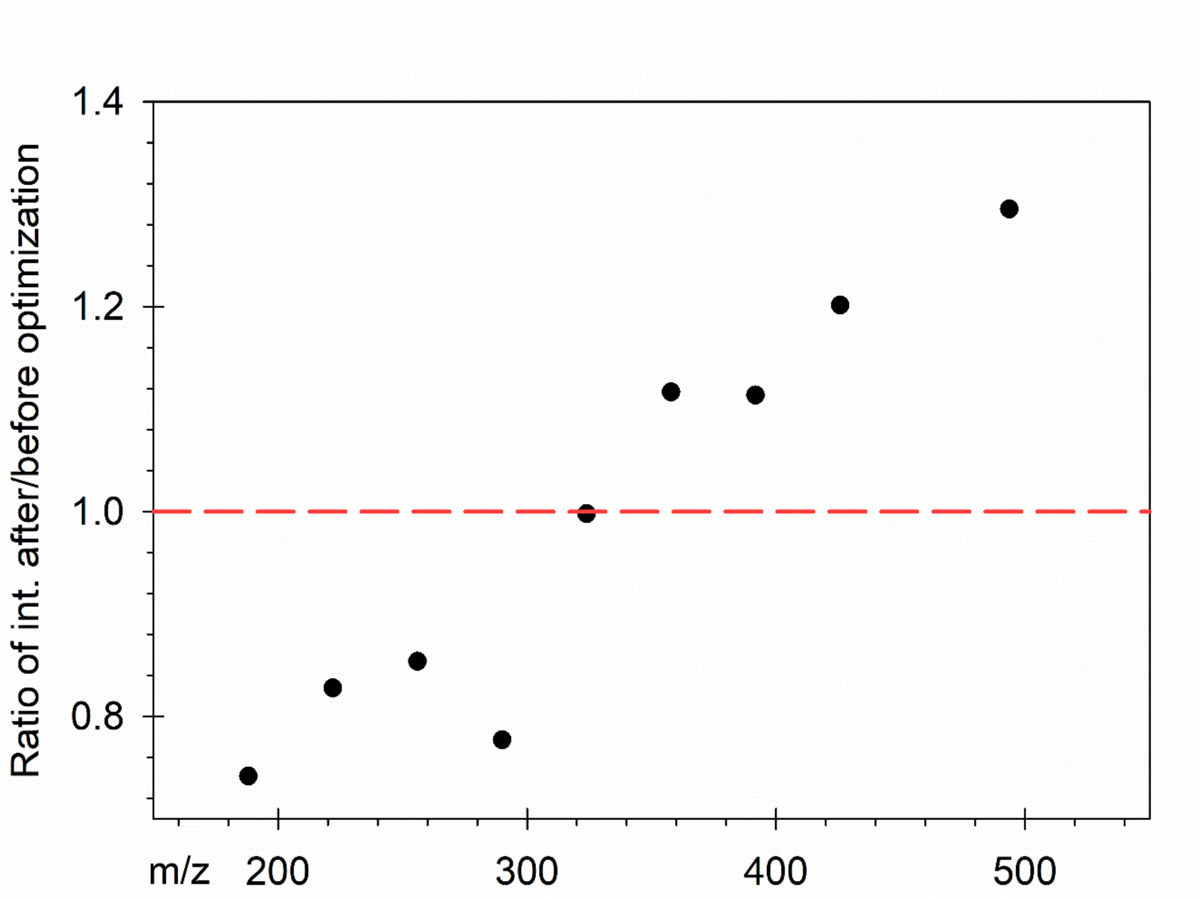


**Figure S5. Intensity fold changes for polychlorinated biphenyls using optimized and default tuning parameters.**

**Section B. Sampling procedure and analysis of total organic carbon.**

Nine surface sediment samples (i.e., Baltic1-9) were collected from the Baltic Sea using a bottom sled in January 2023 and 2024.[2] The samples were sieved (mesh size 1 mm) to remove macrofauna and placed in coolers for transportation. For total organic carbon (TOC) analysis, sediment samples were dried, homogenized, and analyzed using the Flash 2000 (Thermo Scientific).

Two Arctic shelf surface sediment samples (YS-37 and YS-40) were collected during August/September 2008 as part of the International Siberian Shelf Study 2008 (ISSS-08) onboard the H/V Yakob Smirnitskiy. Detailed procedures were described in Vonk et al.[3] Briefly, surface sediments were collected using a Van Veen grab sampler (GEMAX, Kart Oy, Finland; modified at Stockholm University). The surface layers of the grab samples were retrieved with stainless steel spatulas and transferred into polyethylene containers. After freeze-dried, samples were kept at room temperature. TOC was measured on triplicates using an elemental analyzer isotope ratio mass spectrometer (Europe Hydra 20/20, University of California, Davis Stable Isotope Facility, USA).

A lake surface sediment sample (NL) was collected from Lake Tyrifjorden, Norway, a freshwater lake with factories, wastewater treatment plants, landfills, and fire stations on the banks of major inflowing rivers. Three cores were collected from a 10 $m^2$ area near the river mouth in October, 2021, using a Kajak-Brinkhurst sediment corer. Cores were sliced every 2 cm, and layers at the same depths were pooled. The uppermost layer was analyzed in this study. The TOC was determined using thermal oxidation and infrared detection by ALS Laboratory Group AS, following CSN ISO 10694 and CSN EN 13137:2002. Details can be found in Langberg et al.[4]

**Table S2. Sampling and total organic carbon content (TOC) information.**

| Samples | Region | Sediment depth (cm) | Water depth (m) | Latitude (º N) | Longitude (º E) | Sampling date | TOC (%) |
|---|---|---|---|---|---|---|---|
| Baltic1 | Baltic Sea | 0-2 | 25 | 58.26 | 16.91 | Jan. 2023 | 1.6 |
| Baltic2 | Baltic Sea | 0-2 | 36 | 58.79 | 17.73 | Jan. 2023 | 2.7 |
| Baltic3 | Baltic Sea | 0-2 | 10 | 56.08 | 14.94 | Jan. 2024 | 7.7 |
| Baltic4 | Baltic Sea | 0-2 | 28 | 56.08 | 14.98 | Jan. 2024 | 5.8 |
| Baltic5 | Baltic Sea | 0-2 | 18 | 56.07 | 14.97 | Jan. 2024 | 6.2 |
| Baltic6 | Baltic Sea | 0-2 | 17 | 56.07 | 14.96 | Jan. 2024 | 6.1 |
| Baltic7 | Baltic Sea | 0-2 | 28 | 56.07 | 17.81 | Jan. 2023 | 2.2 |
| Baltic8 | Baltic Sea | 0-2 | 43 | 58.52 | 17.32 | Jan. 2024 | 1.8 |
| Baltic9 | Baltic Sea | 0-2 | 42 | 58.78 | 17.69 | Jan. 2024 | 2.4 |
| YS-37 | East Siberian Sea | 0-1 | 42 | 70.14 | 168.01 | Aug./Sep. 2008 | 0.94 |
| YS-40 | East Siberian Sea | 0-1 | 49 | 71.48 | 170.55 | Aug./Sep. 2008 | 1.4 |
| NL | Lake Tyrifjorden, Norway | 0-2 | 5 | 60.03 | 10.17 | Oct. 2021 | 4.5 |

**Section C. Sample preparation.**

Extraction was performed using an accelerated solvent extraction system (ASE 350; Dionex, U.S.A). 34-mL extraction cells were pre-cleaned using 20 mL of acetone/n-hexane (ACE/HEX 1:1 v/v) at 100 °C for 10 min. Empty cells were dried in a clean fume hood at room temperature overnight. Cells were loaded sequentially with a pre-baked glass-fiber filter (GFF; Dionex, Thermo Scientific), diatomaceous earth (DE; Dionex, Thermo Scientific), approximately 4 g of dry sediment, DE, and a GFF. Sediment was fortified with labeled internal standards. DE was used as received. Samples were extracted three times, each time with 20 mL of ACE/HEX (1:1 v/v) at 100 °C for 10 min. The system was washed three times with 5 mL of solvent each time between two samples. Extracts were concentrated to ~1 mL using a rotary evaporator at 30 °C, and transferred to a pre-baked test tube. Between two samples, the connector of the rotary evaporation system was rinsed three times, each time with 1 mL of ACE. 1 mL of active copper in HEX was added to remove sulfur. After vortexing and centrifugation, supernatant was collected into a clean brown bottle, blew down with nitrogen to 200 μL, and transferred to a sample vial. All glassware and quartz filters were burned at 450 °C for 4 h, and metal parts were ultrasonicated for 20 min using ACE. Except for PEEK seals for ASE and caps for sample vials, other plastic items were not used for sample preparation. To prevent potential photolysis, containers were either wrapped in with aluminum foil or amber glassware was used.

**Section D. Instrumental methods of GC-low resolution MS.**

Chromatographic parameters for low-resolution systems were similar to those of GC-APCI-IM-HRMS. For the EI instrument, 1 μL sample was injected in programmed temperature vaporization mode. The inlet temperature was initially set at 70 °C and increased to 300 °C at 900 °C $min^{-1}$. Analytes were separated using 30-m TG-5SILMS columns (0.25 mm i.d., 0.25 μm film thickness; Thermo Scientific, U.S.A). The helium carrier gas flowed at a constant rate of 1.5 mL $min^{-1}$. The oven temperature program was identical to the high-resolution system. The transfer line was maintained at 290 °C. The MS was operated in SIM mode with the ion source temperature at 250 °C.

For the ECNI instrument, 1 μL sample was injected in solvent vent mode. The inlet temperature was initially set at 100 °C for 0.15 min, increased at 600 °C $min^{-1}$ to 280 °C, and held for 1 min with helium carrier gas at a flow rate of 1.5 mL $min^{-1}$. Analytes were separated using a 30-m DB-5MS Ultra Inert column (i.d., 0.25 mm; film thickness, 0.25 μm; Agilent Technologies). The oven temperature program was identical to the high-resolution system. The transfer line, ion source, and quadrupole were maintained at 290 °C, 300 °C, and 150 °C, respectively. The ammonia was used as reagent gas at 80% flow.

**Section E. Software parameters.**

Progenesis QI (version 3.0, Waters Corp., Wilmslow, U.K.) was employed for peak-picking and alignment, with the sensitivity threshold of 50. To simplify data, all ions at the $MS^1$ level were considered as $M^{+\cdot}$. At the $MS^2$ level, fragment ions with intensities lower than 1% of parent ions were excluded. Based on our current computer setup (i.e., Intel® Xeon® W-2125 CPU at 4.00 GHz, an x64architecture, and 64 GB of physical memory), it took ~10 minutes to process one raw data, before $MS^1$ and $MS^2$ data can be exported from Progenesis QI. $MS^1$ is exported as an excel file, containing exact mass, CCS, RT, and area, while $MS^2$ is exported as a TXT file in the mass spectra library format.

SIRIUS+CSI:FingerID (version 5.8.6) was used to estimate $MS^2$ similarity.[5] For APCI, analytes can generate $M^{+\cdot}$ or $[M+H]^+$ through charge/proton transfer, respectively. Although either adduct form can be chosen, the parent ion in SIRIUS has to be the protonated form. Therefore, a mass of hydrogen (1.0078 Da) was added to each isotope of $M^{+\cdot}$ at the $MS^1$ level, before input. Since candidates were known after exact mass, RT, and CCS match, we specified the formula, adduct, parent ion, and InChIKey. Default values were used for other parameters.

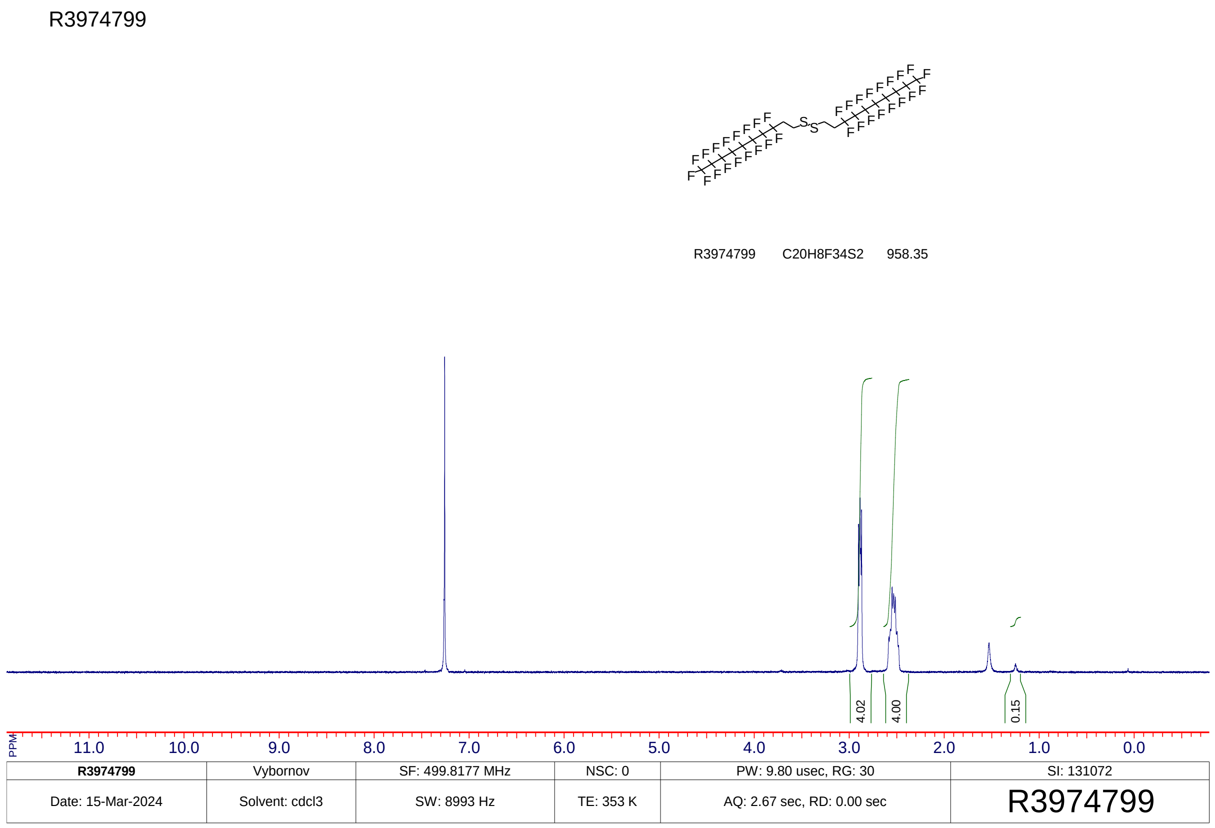


**Figure S6. $^1$H NMR spectrum of 8:2/8:2 fluorotelomer disulfide.** The spectrum is provided by AKos GmbH (Lörrach, Germany).

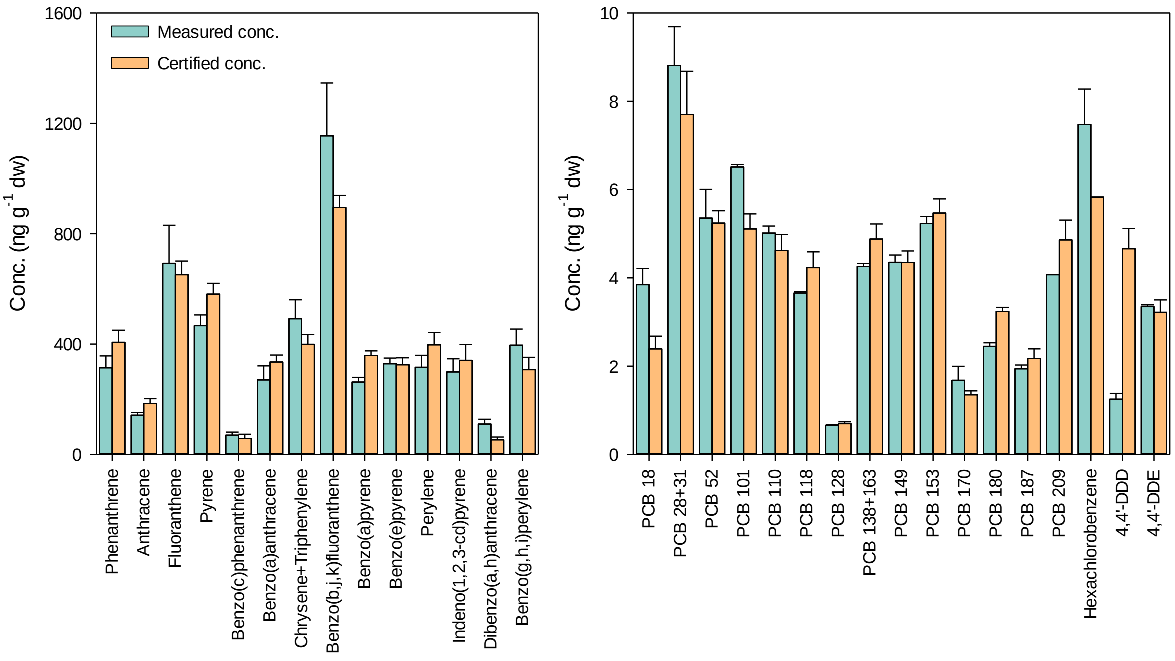


**Figure S7. Comparison of measured and certified concentrations of contaminants in standard reference sediment 1941b.** Concentrations of unseparated compounds were combined. PCB: polychlorinated biphenyl; DDD: dichlorodiphenyldichloroethane; DDE: dichlorodiphenyldichloroethylene.

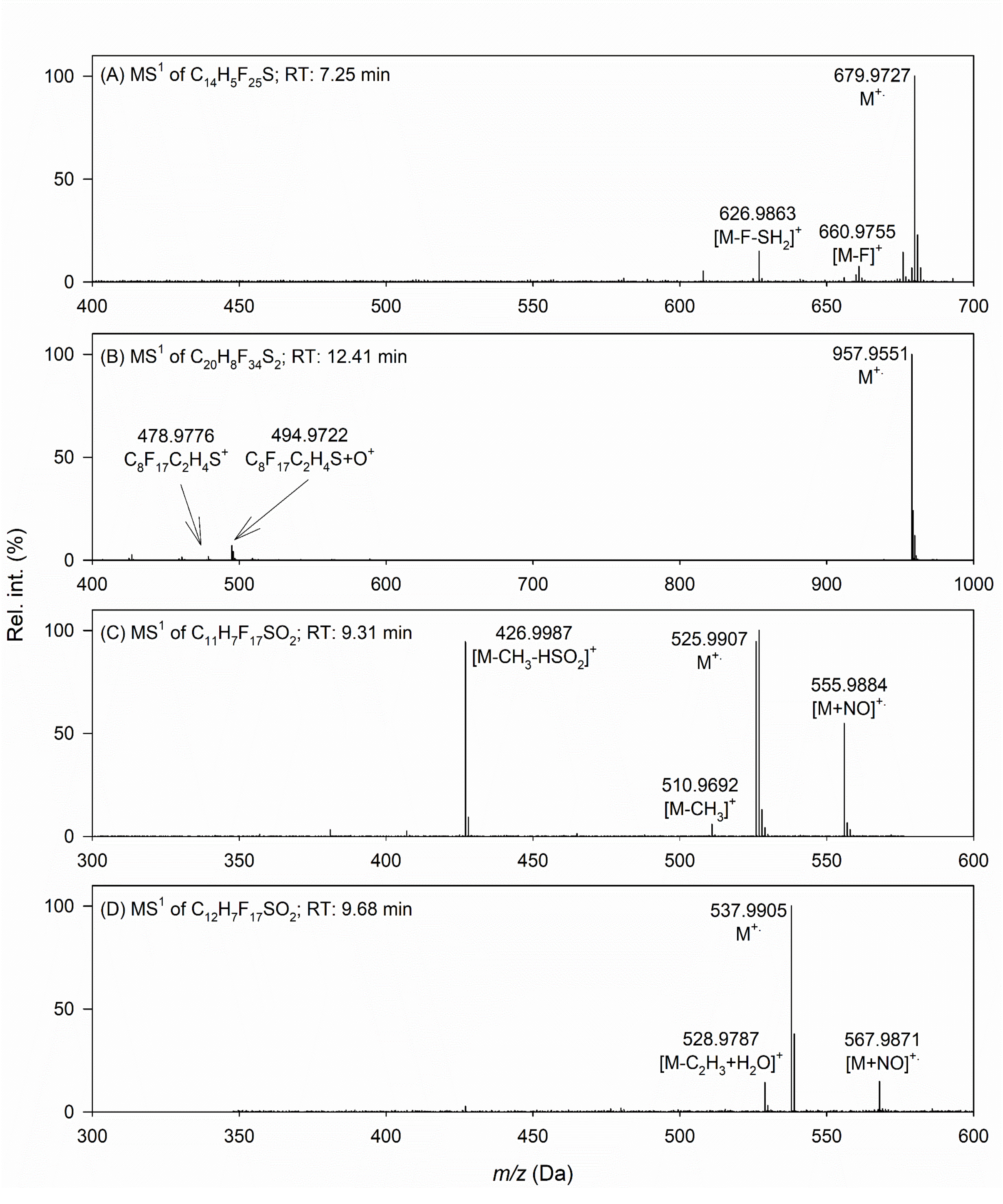


**Figure S8. Full scan mass spectra (MS[1]) of (A) $C_{14}H_5F_{25}S$ at retention time (RT) 7.25 min, (B) $C_{20}H_8F_{34}S_2$ at RT 12.41 min, (C) $C_{11}H_7F_{17}SO_2$ at RT 9.31 min, and (D) $C_{12}H_7F_{17}SO_2$ at RT 9.68 min.**

## References


(1) Waters. Hints&tips guidance for users. Document number: 720005739.

(2) Blomqvist, S.; Lundgren, L. A benthic sled for sampling soft bottoms. *Helgoländer Meeresunters.* **1996**, *50*, 453–456.

(3) Vonk, J.; Sánchez-García, L.; van Dongen, B.; Alling, V.; Kosmach, D.; Charkin, A.; Semiletov, I.; Dudarev, O.; Shakhova, N.; Roos, P.; Eglinton, T.; Andersson, A.; Gustafsson, Ö. Activation of old carbon by erosion of coastal and subsea permafrost in Arctic Siberia. *Nature.* **2012**, *489*, 137–140.

(4) Langberg, H.; Arp, H.; Breedveld, G.; Slinde, G.; Høiseter, Å.; Grønning, H.; Jartun, M.; Rundberget, T.; Jenssen, B.; Hale, S. Paper product production identified as the main source of per- and polyfluoroalkyl substances (PFAS) in a Norwegian lake: Source and historic emission tracking. *Environ. Pollut.* **2021**, *273*, 116259.

(5) Dührkop, K.; Fleischauer, M.; Ludwig, M.; Aksenov, A.; Melnik, A.; Meusel, M.; Dorrestein, P.; Rousu, J.; Böcker, S. SIRIUS 4: A rapid tool for turning tandem mass spectra into metabolite structure information. *Nat. Methods.* **2019**, *16*, 299–302.
